# Deploying a Proximal Sensing Cart to Identify Drought-Adaptive Traits in Upland Cotton for High-Throughput Phenotyping

**DOI:** 10.3389/fpls.2018.00507

**Published:** 2018-04-23

**Authors:** Alison L. Thompson, Kelly R. Thorp, Matthew Conley, Pedro Andrade-Sanchez, John T. Heun, John M. Dyer, Jeffery W. White

**Affiliations:** ^1^U.S. Arid Land Agricultural Research Center, United States Department of Agriculture, Agricultural Research Service, Maricopa, AZ, United States; ^2^Department of Agriculture and Biosystems Engineering, Maricopa Agricultural Research Center, The University of Arizona, Maricopa, AZ, United States

**Keywords:** high-throughput phenotyping, proximal sensing carts, upland cotton (*Gossypium hirsutum* L.), abiotic stress, plant breeding

## Abstract

Field-based high-throughput phenotyping is an emerging approach to quantify difficult, time-sensitive plant traits in relevant growing conditions. Proximal sensing carts represent an alternative platform to more costly high-clearance tractors for phenotyping dynamic traits in the field. A proximal sensing cart and specifically a deployment protocol, were developed to phenotype traits related to drought tolerance in the field. The cart-sensor package included an infrared thermometer, ultrasonic transducer, multi-spectral reflectance sensor, weather station, and RGB cameras. The cart deployment protocol was evaluated on 35 upland cotton (*Gossypium hirsutum* L.) entries grown in 2017 at Maricopa, AZ, United States. Experimental plots were grown under well-watered and water-limited conditions using a (0,1) alpha lattice design and evaluated in June and July. Total collection time of the 0.87 hectare field averaged 2 h and 27 min and produced 50.7 MB and 45.7 GB of data from the sensors and RGB cameras, respectively. Canopy temperature, crop water stress index (CWSI), canopy height, normalized difference vegetative index (NDVI), and leaf area index (LAI) differed among entries and showed an interaction with the water regime (*p* < 0.05). Broad-sense heritability (*H*^2^) estimates ranged from 0.097 to 0.574 across all phenotypes and collections. Canopy cover estimated from RGB images increased with counts of established plants (*r* = 0.747, *p* = 0.033). Based on the cart-derived phenotypes, three entries were found to have improved drought-adaptive traits compared to a local adapted cultivar. These results indicate that the deployment protocol developed for the cart and sensor package can measure multiple traits rapidly and accurately to characterize complex plant traits under drought conditions.

## Introduction

Field-based high-throughput phenotyping (FB-HTP) is a novel approach to characterize complex traits in large plant populations using proximal and remote sensing or imaging. The power of high-throughput phenotyping (HTP) is its ability to characterize plant traits for large populations in both time and space, which improves monitoring of dynamic genetic responses to environmental conditions. The development of HTP for use in plant breeding and other programs has steadily increased over the last 10 years. Evaluated platforms include simple manual push carts (White and Conley, 2013), tractors (Comar et al., 2012; Andrade-Sanchez et al., 2014; Deery et al., 2014; Barker et al., 2016), field-scanners (Virlet et al., 2017), and unmanned aerial systems (UASs) (Rutkoski et al., 2016; Yu et al., 2016). Sensor packages have included non-contact spectral reflectance and acoustic sensors (Montes et al., 2007, 2011; Andrade-Sanchez et al., 2014; Thorp et al., 2015b), red, green, blue (RGB) digital cameras (Mendes et al., 2016; Thorp et al., 2016; Yu et al., 2016), 3D imagers based on light detection and ranging (LiDAR) (Sirault et al., 2013; Lin, 2015; French et al., 2016), and multispectral cameras (Rutkoski et al., 2016; Virlet et al., 2017), among others. The ideal platform and sensor package for a breeding program greatly depends on program parameters and available resources, including funding, access to reliable GPS equipment and signal correction services, personnel that can utilize the technology and corresponding software, target crop(s), number/diversity of field sites, and traits under evaluation.

Proximal Sensing Carts (PSCs) are a low-cost option for breeding programs that aim to incorporate proximal sensors or cameras for HTP. PSCs are typically lightweight, narrow-wheeled and relatively small, cause minimal soil compaction with repeated sampling, and are easy to transport to multiple fields (White and Conley, 2013). Hand pushed or motorized PSCs can be designed to fit specific crop height and row spacing requirements, while deploying multiple sensors (White and Conley, 2013; Bai et al., 2016). The PSCs deployed by White and Conley (2013) and Bai et al. (2016) were equipped with modular sensor packages including thermal infrared radiometers (IRTs) for measurement of canopy temperature and RGB cameras for estimating canopy cover. Bai et al. (2016) also incorporated ultrasonic displacement sensors and spectral reflectance sensors as part of their sensing package, and other researchers have used similar equipment for proximal sensing on PSCs and other FB-HTP platforms (Comar et al., 2012; Andrade-Sanchez et al., 2014; Deery et al., 2014; Barker et al., 2016). Well-designed and constructed PSCs can enable the rapid and simultaneous collection of multiple traits associated with heat and drought stress across multiple environments and crops at reduced cost or time compared to tractor or UAS platforms. However, additional research is needed to understand whether data collected using the PSC platforms can assist breeding decisions and contribute to identification of improved genotypes as reported by the tractor or UAS platforms.

A FB-HTP “tri-metric” sensor package, including infrared thermometers, active spectral reflectance, and ultrasonic displacement sensors, is particularly useful for measuring heat and drought stress responses in field-grown crops. Including a digital RGB camera offers further opportunities for phenotyping via image analysis. Canopy temperature, as measured by infrared thermometry, has long been recognized as a powerful indicator of plant stress under water deficit (Jackson et al., 1981; Howell et al., 1984; Fuchs, 1990). Reduced canopy temperatures are correlated with increased yield in wheat (*Triticum aestivum*) (Amani et al., 1996), cotton (*Gossypium hirsutum* L.) (Pauli et al., 2016), and other crops (Chavez et al., 2002). Reduced crop canopy temperature is also associated with improved stomatal regulation by abscisic acid signaling (ABA) (Shinozaki and Yamaguchi-Shinozaki, 2007; Pauli et al., 2016) and increased root biomass (Lopes and Reynolds, 2010).

The utility of spectral reflectance sensors to examine plant and soil traits across environments is also well documented. Many spectral indices, including the normalized difference vegetation index (NDVI), have been developed to estimate biomass, leaf area index (LAI), and chlorophyll from spectral reflectance measurements of crop canopies (Haboudane et al., 2004; Montes et al., 2011; Gutierrez et al., 2012; Mulla, 2013; Thorp et al., 2015a). Abiotic stress during early canopy development can decrease plant biomass and height, reduce leaf area, and abbreviate green area duration (Araus et al., 2002). In cotton, NDVI measurements at peak flower were associated with 47% of the variation in lint yield (Gutierrez et al., 2012). While not identified as the best index to use for prediction of lint yield, the results suggested that NDVI is a useful indicator of drought-associated changes in crop canopy architecture, which subsequently affects crop yield. Used together, IRTs, multi-spectral reflectance, ultrasonic sensors, and RGB cameras can detect spatial and temporal plant responses to heat and drought stress, which can assist germplasm selection and identification of genes that underpin these complex traits (Pauli et al., 2016; Rutkoski et al., 2016).

Since White and Conley (2013) described the initial PSC for FB-HTP in Arizona, the team has continued to improve and test PSC designs, resulting in several new PSC prototypes. The primary goal of the present study was to assess performance of a novel PSC with a specific deployment protocol for crop improvement research, using cotton as a test case. The objectives were to (i) describe the design and implementation of a custom PSC that improves on previous PSC designs (ii) develop a deployment protocol for the PSC in a cotton breeding trial under well-watered and water-limited treatments and (iii) illustrate the use of proximal sensing data to identify lines with drought-adaptive traits.

## Materials and Methods

### Proximal Sensing Cart (PSC) and Phenotyping System

**FIGURE 1:**
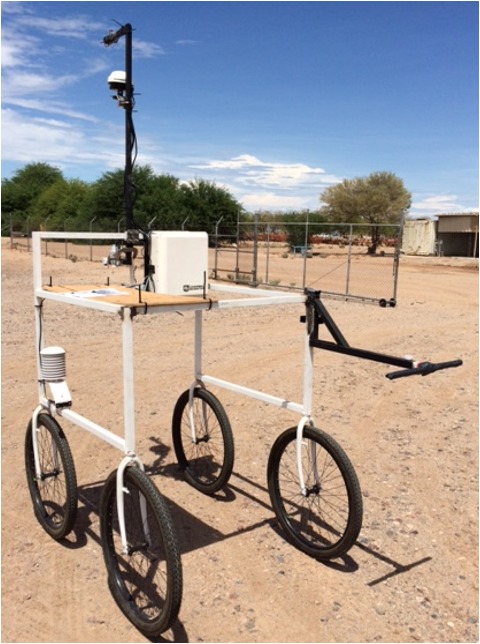
Proximal sensing cart with a sensor bar, data logger, sensors, and extended handle. Camera sensor bar and cameras are not shown.

The PSC developed for this study (**Figure 1**) was designed using similar materials and fabrication protocols as reported by White and Conley (2013). The frame was constructed with 3.2 cm square steel tubing. The front frame was approximately 34 cm × 105 cm × 99 cm (l × w × h), which supported the sensor package and data logger. The frame was attached to four standard 61-cm bicycle forks, which were fixed for straight tracking and pivot turning. Approximately 5 cm of the bicycle frame was inserted into the square tubing before welding to reinforce the union point. A removable, extended handle was provided for ease of pushing and maneuvering in the field. The handle was L-shaped with the short end fitting inside the frame, and extended 122 cm from the main body of the PSC. A secondary bar was added perpendicular to the 90° angle to reinforce the handle when lifting the rear wheels to turn the cart. Frame components were welded using a wire-feed MIG welder (Lincoln Electric, Cleveland, OH, United States). A scaled rendering of the PSC design is provided in **Supplementary Figure S1**.

The sensing package deployed on the PSC included the core “tri-metric” sensor package (described below) for measuring canopy height, canopy temperature, and canopy reflectance; a modified weather station for measuring ambient air temperature, relative humidity, and incoming radiant energy; a GPS receiver; an altitude heading and reference system (AHRS) that included an inertial measurement unit (IMU); and RGB cameras. A sensor arm bar, made from 3.2-cm square tubing in a C-shape was anchored to the front frame using U-bolts. The sensor bar can be raised or lowered by loosening the U-bolts and sliding the bar to a desired height. A Campbell Scientific (Logan, Utah) CR1000 data logger and enclosure were anchored to the front of the frame. The camera arm consisted of a simple 5 cm × 5 cm square wooden stick clamped to the top of the cart frame and extended 61 cm beyond the end of the sensor bar. For easy adjustment and removal, sensors were attached using industrial dual lock fastener tape (3M Scotch Brand), zip ties, or U-bolts.

Canopy height (mm) was measured with an ultrasonic transducer (UC2000-30GM-IUR2-V15, Pepperl+Fuchs, Twinsburg, OH, United States) that had a sensing range between 80 and 2000 mm and a narrow field of view (FOV) (25° half-angle) operating at 180 kHz frequency. The analog sensor output was converted to displacement values after applying the calibration equation supplied by the manufacturer. The sensor was attached to the sensor bar with a U-bolt and oriented vertically downward (nadir view).

Canopy temperature (°C) was measured with an infrared thermometer (IRT) sensor (SI-131, Apogee Instruments, Logan, UT, United States) with a narrow FOV (14° half-angle). The sensor output (μV) was converted to temperature values after applying a polynomial calibration equation supplied by the manufacturer. The IRT sensor was mounted in an insulated shroud, attached to the sensor bar with a U-bolt, and oriented vertically downward (nadir).

Canopy reflectance (ρ) was measured with passive spectral reflectance sensors (SRS NDVI, Decagon Devices, Pullman, WA, United States). The uplooking (zenith) sensor had a hemispherical view and was mounted at the top of the sensor bar. The downlooking (nadir) sensor had a 36° FOV and was mounted at the bottom of the sensor bar. The sensor measured light reflectance in one visible (VIS) and one near infrared (NIR) waveband, centered at 650 nm and 810 nm with 50 nm and 40 nm full width at half maximum, respectively. The sensor outputs for both wavebands calculated normalized difference vegetative index (NDVI) as follows:

$$NDVI\;=\;\frac{\left(\rho NIR-\rho VIS\right)}{\left(\rho NIR\;+\;\rho VIS\right)}$$

Weather data were collected in real-time with a pyranometer for radiant energy (RAD) (SP-110, Apogee Instruments, Logan, UT, United States) and a probe for air temperature and relative humidity (Rh) (HC2S3, Campbell Scientific Logan, UT, United States). The pyranometer had a 180° FOV with a spectral range from 360 to 1120 nm. The pyranometer was mounted at the top of the sensor bar with industrial fastener tape and oriented in the vertical direction pointing upward (zenith). The HC2S3 probe had two parts, a PT1000 resistance temperature detector (RTD) that measured air temperature at ±0.1°C accuracy and a rotronic hygrometer that measured relative humidity at ±0.8% RH accuracy. This sensor was mounted inside the cart frame on the front left wheel support bar and close to the canopy.

Measurements from each sensor were georeferenced by simultaneously recording position from a Global Position Satellite (GPS) receiver (A101 Smart Antenna, Hemisphere GPS, Scottsdale, AZ, United States) using the GGA and RMC NMEA messages, and heading from an inertial (IMU) sensor (VN-100, VectorNav Technologies, LLC, Dallas, TX, United States). The GPS receiver was mounted halfway up the sensor arm with a U-bolt while the IMU sensor was fastened to the logger enclosure. Sensor information was logged at a rate of 5 Hz (except for the SRS); 0–5 V analog signals and serial (RS232) data messages were captured on a data logger (CR1000, Campbell Scientific, Logan UT, United States). The digital SRS was logged at 0.5 Hz using SDI12 communication protocol on the same data logger.

Images were captured using either a Garmin VIRB XE 12.4 MP action camera with internal GPS and metadata (Garmin Ltd., Olathe, KS, United States) or a Canon PowerShot SX600 HS with 16.8 MP (Canon Inc., Melville, NY, United States). Cameras were mounted to the end of the camera bar using industrial fastener tape with a nadir view over one row of each plot. Images were stored in JPEG format.

### Field Experimental Design and Irrigation Management

A cotton field trial was conducted in 2017 at the University of Arizona, Maricopa Agricultural Center (33.068°N, 111.971°W, 360 m elevation), in Maricopa, AZ, United States. Maricopa is located in an irrigated production area of the low desert that receives less than 100 mm of rainfall during the cotton growing season (April–September). Air temperatures ranged from 11°C nighttime lows to 48°C daytime highs during the cotton season. The soil type is a Casa Grande sandy loam (fine-loam, mixed, superactive, hyperthermic Typic Natrargids). The field was divided into six irrigation borders (basins) with 12 columns per border; each border was 118.5 m long and 12.2 m wide. Thirty upland cotton (*G. hirsutum* L.) elite breeding lines and five commercial checks (**Table 1**) were grown under well-watered (WW) and water-limited (WL) conditions using a (0,1) alpha lattice design with three replications per treatment. Experimental plots included two 12.1 m cotton rows with 1.02 m inter-row spacing and a density of approximately 8.6 plants m^-2^. There was a 1.5 m alley between adjacent plots on the same row. Plot boundaries were geospatially delineated using a geographic information system (ArcGIS v. 10.2, ESRI, Redlands, CA, United States) to create a plot polygon map in shapefile format prior to planting. The eastern and western most rows of each border were planted with a commercial cotton variety (DP1549B2XF, Monsanto, St. Louis, MO, United States) to reduce edge effects. Likewise, a 2.02 m buffer of the commercial variety was planted along the north and south ends of each border (**Supplementary Figure S2**).

**Table 1:** Cotton entries provided by the Regional Breeders Testing Network, listed by the program entry number with the corresponding breeding line name and originating state.

Entry Number	Entry name	State
1	LA14063046	Louisiana
2	LA14063101	Louisiana
3	LA14063038	Louisiana
4	LA14063001	Louisiana
5	LA14063083	Louisiana
6	TAM 13S-03	Texas
7	TAM WK-11L	Texas
8	TAM 13Q-51	Texas
9	Tamcot G11	Texas
10	TAM 13Q-18	Texas
11	PD 2013016	South Carolina
12	PD 07040	South Carolina
13	PD 08028	South Carolina
14	PD 09084	South Carolina
15	PD 09046	South Carolina
16	Ark 0921-27ne	Arkansas
17	Ark 0912-18	Arkansas
18	Ark 0921-31ne	Arkansas
19	Ark 0911-13	Arkansas
20	Ark 0908-60	Arkansas
21	NM 16-13P1088B	New Mexico
22	NM 13R1015	New Mexico
23	Acala 1517-08	New Mexico
24	TAM LBB130218	Texas
25	TAM LBB131001	Texas
26	AU 90098	Alabama
27	GA 2012141	Georgia
28	GA 2015032	Georgia
29	GA 2015073	Georgia
30	GA 2015090	Georgia
31	DP393	Check
32	DP493	Check
33	FM958	Check
34	UA222	Check
35	DP1549B2XF	Local Check

Plots were planted on May 10, 2017. To germinate seed and establish plants, the field was furrow flooded. On June 01, 2017, the irrigation method was switched to microirrigation via buried drip tape (Netafim, Fresno, CA, United States), which was installed at a 20 cm depth prior to planting. Irrigations were scheduled from a daily crop water use and soil water balance model based on Food and Agricultural Organization-56 (FAO-56) methods (Allen et al., 1998; Hunsaker et al., 2005). Meteorological data were obtained from the Arizona Meteorological Network (AZMET) weather station approximately 280 m from the center of the field site^1^. The water-limited treatment reduced irrigation applications to 70% of the recommended amount beginning on July 12, 2017 after approximately 50% of plants within all plots had flowered. Prior to this date, all plots received equal irrigation at 100% of the recommended amount. Soil moisture was monitored weekly using a neutron moisture meter (503E, InstroTek Inc., San Francisco, CA, United States) with measurements collected every 20 cm to a depth of 2 m within 5 mm steel access tubes installed after crop emergence. Each border contained three access tubes for soil moisture measurements, which were evenly distributed from north to south.

### Physiological and Growth Stage Notes

Four weeks after emergence, seedlings were counted in each row of each plot to calculate the plant density. The dates on which 50% of plants per plot showed first square (flower bud) and first flower were recorded. After irrigation treatments were established, pollen sterility was recorded on a scale of 1–5 from five representative flowers per plot (July 27): “1” meant no observed sterility and “5” meant complete sterility.

### Proximal Sensing Cart (PSC) Field Deployment

The PSC was deployed on June 16 (DOY 167), June 30 (DOY 181), July 14 (DOY 195), and July 28 (DOY 209). Collection start times were 09:15 am, 10:11 am, 09:25 am, and 09:32 am, respectively. Prior to the start of each collection, the sensor bar was adjusted to 40 cm above the crop. The distance from soil line to sensor bar and from plant terminal bud to sensor bar were measured and recorded as “metanotes” over representative plants from a single plot. The representative plants were also manually measured for canopy height (cm) and canopy temperature (°C) at the start and stop of each collection. The heights were measured with a standard 1 m ruler from the soil line to the terminal bud, and canopy temperature was measured with a handheld IRT at the same height as the sensor bar (MI-2K0, Apogee Instruments, Logan, UT, United States). The soil temperature was measured using the handheld IRT in the non-shaded furrow next to the representative plant samples. To maintain a consistent cart speed, the stopwatch application on a cellular phone (iPhone 6, Apple Inc., Cupertino, CA, United States) was used to maintain a manual pushing pace of ∼2 min per 118.5 m row length.

To improve data quality with the proximal sensors, anchor targets were deployed in the first and third row of each irrigation border in the alley between the southernmost plots and the northernmost plots. The first target, to the south, was a 9-L clear storage container (20.0 cm × 36.8 cm × 15.9 cm) filled with ice water for assessing IRT measurements. The ice bucket was maintained at 0–4°C during data collection as verified by a handheld IRT measurement 5 cm above its surface. The second target, to the north, was an empty white Styrofoam box (26 cm × 26 cm × 20 cm) used to establish a white color for assessing the spectral reflectance sensor data and known height above the planted beds for assessing the ultrasonic displacement sensor. As the target locations were geospatially known, they also provided a visual and physical “anchor” to validate the geospatial processing (explained below). The two targets for spatially binning the RGB cameras included the orange stakes used as plot markers at the southwestern corner of each plot and the red caps of the access tubes used for soil moisture measurements. For the first collection, only the VIRB camera was operational, and no images were captured on the final collection day.

### Management of PSC Collected Data

The PSC data were transferred from the Campbell data logger to a server designated for FB-HTP. The Campbell data logger SD card was removed and inserted into a card reader; data were then converted from binary to comma-delimited text files at the server terminal. A similar process was used for the cameras and captured images. The server was a dual Intel xenon 12-core processor with the Windows Server 2012 R2 operating system. The server had 256 GB of memory, 260 TB of storage and an NVidia K80 Tesla graphics card. Files were organized first by field name, year of collection, day of collection, and then sensor type (proximal or RGB). All files followed this naming scheme for downstream data processing and analysis. Three sensor files were transferred from the Campbell data logger: (1) the main file which contained the thermal, height, and weather data keyed with the GPS and ARHS data by the logger timestamp; (2) the NDVI SRS reflectance data keyed with the logger timestamp; and (3) a file with all text strings from the GPS and ARHS with custom error codes and logger timestamp. The NDVI data were merged with the GPS and ARHS by matching timestamps in post processing.

### Geospatial Data Processing of PSC Collected Phenotypes

Two main geoprocessing steps were needed to prepare the data for analysis: (1) “georeferencing” the sensor data by assigning geospatial coordinates to each sensor measurement and (2) matching sensor data to plot information using geographic information system (GIS) tools to locate sensor data within plot boundaries. To georeference the sensor data, the vehicle position, vehicle heading, and the offset distances from the GPS receiver to each sensor were required. The GPS data provided latitude and longitude coordinates (*X* and *Y*) of the GPS receiver position at each logger timestamp, while the AHRS-derived yaw measurement provided information about the orientation of the cart on the Earth’s surface (i.e., the heading angle of the vehicle). In addition, both the forward and lateral distances from the GPS antenna to the location of each sensor were obtained from the design specifications of the PSC. To facilitate geospatial data analysis based on a two-dimensional planar coordinate system, the latitude and longitude coordinates (units of decimal degrees) were projected to the Universal Transverse Mercator (UTM) coordinate system (units of meters). Projections were based on the World Geodectic System (WGS) 1984 (WGS-84) datum. The field site was located in UTM zone 12N. Using a set of trigonometric equations (Wang et al., 2016) for coordinate system transformations, the UTM position of each sensor measurement was calculated from the vehicle position and heading data and known distances between each sensor and the GPS antenna. Due to error in the Hemisphere GPS positioning measurements, a 1.5 m adjustment to the UTM easting positions was required to align the data with the plot boundary map. The 1.5 m adjustment was determined by comparing the known position of the anchor targets with the calculated UTM coordinates for the target. After UTM coordinates were calculated, each data point located within plot boundaries was annotated using the “Join attributes” function in Quantum GIS (QGIS^2^), which connected information about the plots (e.g., entry and irrigation treatment) with each sensor measurement. Following this step, the georeferenced and geolocated sensor data were exported from QGIS as comma-delimited text files (CSV) for subsequent analysis.

Geospatial data processing was accomplishing using the “HTP Geoprocessor” plugin (Wang et al., 2016) for QGIS. The plugin uses Python scripts to automatically conduct the geoprocessing tasks described previously. An instruction file was created to instruct the GIS how to read data for each sensor type from the logger files. Further detailed instructions on how to use the “HTP Geoprocessor” plugin can be found at http://www.fieldphenomics.org/workshops/2015-htp-workshop under Exercise G (**Supplementary File S1**).

### Statistical Analysis of PSC Derived Phenotypes in Response to Water Treatments

The SAS for Windows software v. 9.3 HPMIXED procedure (SAS Institute, Cary, NC, United States) was used to fit a linear model to each phenotype for outlier removal. Each model included one of the sensed phenotypes [canopy temperature (*Tc*), canopy height (*h*), or NDVI reflectance] as the dependent variable. Canopy height was calculated by subtracting the soil line to sensor bar height from the displacement data measured by the ultrasonic sensor. The cotton entry, irrigation treatment, and the interaction term were modeled as fixed effects; random effects included the replicate nested within treatment and the plot row and column designation nested within border. Outliers were determined by setting an upper and lower limit for the Studentized deleted residuals calculated from the collected data with a criterion of α = 0.05 (Kutner et al., 2004). After outliers were removed, the means for each plot were calculated using the SAS MEANS procedure.

The crop water stress index (CWSI) and LAI were calculated from mean plot data. CWSI was calculated by subtracting the recorded ambient air temperature from the measured canopy temperature (*Tc – Ta*) (Jackson et al., 1981). LAI was calculated using the measured *h* and NDVI output following Thorp et al. (2015a). The plot means for each phenotype were used as the dependent variable for subsequent statistical analysis.

Plant responses to the treatment over time for each of the traits under observation were analyzed by repeated-measures mixed-model analysis of variance using the SAS MIXED procedure. The plot means from each of the four collections for each of the phenotypes (*Tc*, *h*, CWSI, LAI, or NDVI) were used as dependent variables. The cotton entry, irrigation treatment, day of year (DOY), and corresponding interaction terms were fitted as fixed effects. Random effects included the replicate, the treatment-by-DOY-by-replicate interaction, the plot row, column designation nested within border, and the corresponding interactions with replicate, treatment, and DOY. Corrected degrees of freedom were obtained using the “DDFM = KR” option in the model statement. The DOY was included in the REPEATED statement, and a compound symmetry covariance structure (“TYPE = CV” option of the REPEATED statement) was used in the analysis. Least-squares means corresponding to the entry^∗^treatment level of the fixed effects were compared using the “ADJUST = DUNNETT” option of the LSMEANS statement with the DP1549B2XF entry as the control in the corresponding WW or WL treatment.

Broad-sense heritability [*H*^2^ = Var(*G*)/Var(*P*)] on a plot basis, or repeatability, was calculated with the asymptotic variance-covariance matrix (Self and Liang, 1987) for each of the phenotypes from each of the four collections using the plot means as the dependent variable using the SAS code provided by Holland et al. (2003). The overall mean for each phenotype for each DOY was the only fixed effect. Random effects included replicate nested within treatment, entry, and the corresponding interaction. To examine associations among traits, Pearson’s correlation coefficients (*r*) were determined using the SAS CORR procedure.

### Statistical Analysis of Physiological and Growth Stage Notes

Plant physiological data and growth stages were analyzed by mixed-model analysis of variance using the SAS MIXED procedure. The plot means for each of the phenotypes (stand count, first square, first flower, and pollen sterility) were used as dependent variables. The cotton entry, irrigation treatment, and corresponding interaction term were fitted as fixed effects. Random effects included the replicate, nested within border, and the corresponding interaction replicate by treatment. Corrected degrees of freedom were obtained using the “DDFM = KR” option in the model statement. Least-squares means corresponding to the entry level of the fixed effects were compared using the “ADJDFE = ROW” option of the LSMEANS statement.

### RGB Image Analysis for Canopy Cover

Images acquired by the Garmin VIRB were automatically georeferenced with the cameras’ internal GPS and written to the exchangeable image file (EXIF) as metadata. To georeference the images acquired by the Cannon camera, images were spatially binned by manually designating the plot row assignment using the anchor targets. Images were then assigned the plot boundary associated UTM coordinates and ARHS data (yaw, pitch, and roll) by timestamp. Because the timestamp from the camera and Campbell logger were not synchronized, the anchor targets were used to determine the time offset. Images were aligned and orthomosaics were generated using Agisoft Photoscan Professional software v. 1.3.2 (St. Petersburg, Russia) following the online orthophoto protocol with no ground control points (GCP) http://www.agisoft.com/support/tutorials/beginner-level/. The orthomosaic for each row of each plot was exported in standard tagged image file format (tiff) to calculate canopy cover using Fiji Image J v. 1.51n (National Institutes of Health, United States). Originally created in RGB color space, each orthomosaic was then converted to the hue, saturation, and brightness (HSB) color space using the “HSB-Stack” and “Stacks to Images” options in Image J, which produced a 3-band HSB image with 8 bits per band (i.e., HSB was represented with integers from 0 to 255). Segmentation (i.e., spatial partitioning) of plant material in each orthomosaic was accomplished by thresholding the hue band at integer 44: pixel values less than 44 were soil and pixel values greater than 44 were plant. This resulted in a binary image (i.e., a one-band image with pixels equaling either 0 or 255) where “255” represented the green pixels and “0” represented all other pixels in the orthomosaic. The percent canopy cover was initially calculated as the fraction of pixels segmented as green plant material and the total pixels in the orthomosaic using the “Analyze” and “Measure” options in Image J. However, because the orthomosaics focused mainly on the crop row while ignoring the inter-row area, the percent canopy cover calculations were adjusted to reflect the true area of the plot row in cm^2^ based on the area of each pixel as calculated by Agisoft and the known length and width of each plot row. The corrected canopy cover measurements were compared to the other PSC and physiological measurements using the SAS CORR procedure as above. Further detailed instructions on how to calculate canopy cover from an image can be found at http://www.fieldphenomics.org/workshops/2015-htp-workshop under Exercise H (**Supplementary File S2**).

## Results

### Assessed Performance of the PSC and Sensors

**FIGURE 2:**
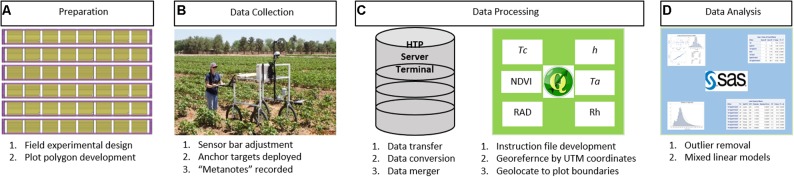
A general workflow for field-based, high-throughput phenotyping using a proximal sensing cart equipped with multiple non-contact sensors and RGB cameras to assess cotton responses to water deficit used in this paper. The workflow starts with experimental and field preparation **(A)** then deployment of the cart for data collection **(B)** followed by data processing **(C)** and ending with data analysis **(D)**. Verbal and written informed consent was obtained for **(B)** from the depicted individual for the publication.

The method workflow for the FB-HTP data acquisition, processing, and analysis is illustrated in **Figure 2**. The field experimental design was planned and polygons to delineate plot boundaries were developed. The PSC and field were prepared for data collection, including sensor bar adjustments, anchor targets, and “metanotes” were recorded. The collected data were transferred from the Campbell logger to the HTP server via the terminal and converted to comma-delimited text files for processing. The data were georeferenced and geolocated within plot boundaries using QGIS and the “HTP Geoprocessor” plugin. Processed data were tested for outliers prior to statistical analysis to determine treatment and entry differences.

In the first two HTP collections, the cart speed was approximately 0.75 m s^-1^ and required just over 2.5 h to cover the 0.87 ha experiment including turns. The last two collections were faster with speeds up to 0.86 m s^-1^, requiring just over 2 h to complete (**Table 2**). The average number of measurements per plot for each sensor after outliers were removed was 123, 122, 128, and 13 for the IRT, displacement, weather station, and SRS, respectively. Total data amounts averaged 50.7 MB per collection. The average number of images per plot was 131. The collected images required 45.7 GB of storage per collection. The amount of image data tripled from Run1 to Run 2 and 3 because both the Cannon and VIRB cameras were operational during Run No. 2 and 3 (**Table 2**).

**Table 2:** Proximal sensing carts (PSC) data collections listed by the run number and day of year (DOY) of the collection.

Run Number	DOY	IRT	ULT	Weather	SRS	Total data (MB)	Images	Total data (GB)	Speed m s^-1^	Start time	Stop time	Total time
1	167	117.5	117.4	139.6	12.7	60.0	116	25	0.731	9:15	11:59	2:44
2	181	139.8	137.0	139.6	14.0	55.8	138	78	0.764	10:11	12:48	2:37
3	195	119.6	118.9	121.9	12.3	45.6	138	72	0.875	9:25	11:42	2:17
4	209	114.6	114.9	110.9	11.8	41.4	n/a	n/a	0.914	9:33	11:44	2:11
Average		122.9	122.1	128.0	12.7	50.7	131	46	0.822	9:36	12:03	2:27

*Included are the average number of data points per plot after outlier detection and removal for the infrared thermometer (IRT), ultrasonic transducer (ULT), weather station, spectral reflectance sensor (SRS), and the corresponding amount of data collected in meta-bytes. Images include those from both RGB cameras and total amount of data collected in giga-bytes. The rate of travel, collection start and stop time and total time of each collection are included.*

To georeference plot measurements, GPS outputs as NEMA RMC and GGA strings were used. In this study, there were intermittent instances where only one of the two NEMA strings was recorded due to logger coding errors. To address the issue, the two NMEA strings were post-processed to select the most appropriate time and positional data which was aided by the use of the anchor targets.

**FIGURE 3:**
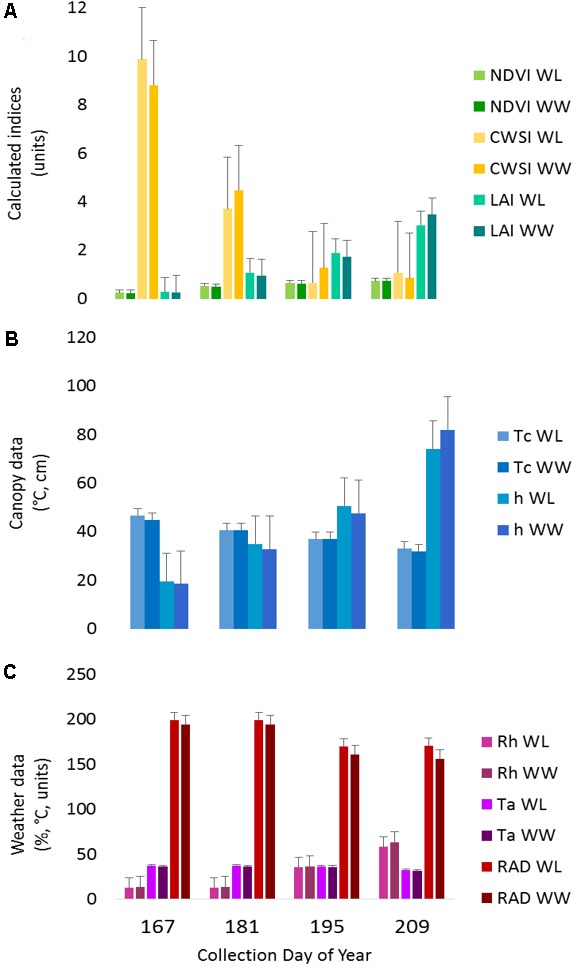
Proximal sensing carts (PSC)-derived phenotypes split out by irrigation treatment for each collection day. The data represent the average of each phenotype for the designated treatment and day of year. **(A)** The normalized difference vegetative index (NDVI), crop water stress index (CWSI), and leaf area index (LAI) data are presented as calculated index units with corresponding error bars. **(B)** The canopy temperature (*Tc*) and canopy height (*h*) with the corresponding error bars. **(C)** The weather data presented in each respective unit of measurement for ambient air temperature (*Ta*), relative humidity (*Rh*), and radiant energy (RAD) with corresponding error bars.

The IRT sensor performed within estimated bounds as established by the hand measurements before and after each run and the target anchor points, except for the first collection. In the first collection, the average *Tc* values were ∼10°C above anticipated values, due to small plant size and soil interference in the sensor field of view. The increased *Tc* influenced the CWSI calculations (*Tc-Ta*), making the plants appear more stressed earlier in the season as compared to the water-limited treatment later in the season. This result is also apparent to a lesser extent in the second collection (**Figure 3**).

The ultrasonic displacement sensor, overall, returned *h* values within the estimated boundaries; however, a negative value would occasionally occur. These values were deemed “impossible” and removed prior to outlier detection and removal. The negative values were attributed to deviations in the plant bed height as compared to the one-time measurement of distance from soil to sensor bar with the metric ruler from the pre-measured soil to sensor bar height. In fact, plant bed heights were not uniform throughout the field, and *h* could vary by 5 cm due to bed height variation that originated at the time of bed shaping. Bed height can continue to vary during the growing season due to soil compaction, rain events, and irrigation leaks that erode the planting beds.

The canopy spectral reflectance sensor (SRS) performed within the manufacturer specifications, and no deviations from the estimated boundaries were detected. Note that considerably fewer measurements were available for this sensor due to the decreased logging rate. As a result, the anchor target was only captured at one–twelfth the rate as the other sensors. The LAI calculations were likely influenced by any deviations in *h* due to bed height variability; however, they fell within the expected range for cotton plants in early-mid season growth (**Figure 3**).

The Garmin VIRB white balance setting was non-functional on the camera used in this study. As a result of the intense sun and changing sun angles found in Maricopa, Arizona, the image gains changed both within and between plots. These gain changes made image alignment and orthomosaic generation difficult; this also prevented consistent threshold values to be set for canopy cover estimates (data not shown). For these reasons, the VIRB images were not used for analysis. The Canon camera produced better images in this environment, but because the camera did not possess an internal GPS, extra steps were required to locate the images within plot boundaries. Due to time restrictions, only the first eight plots (both rows) from the June 30 (DOY 181) collection were fully processed for canopy cover.

### Irrigation Treatments and Sensor “Tri-Metric” Detection

**FIGURE 4:**
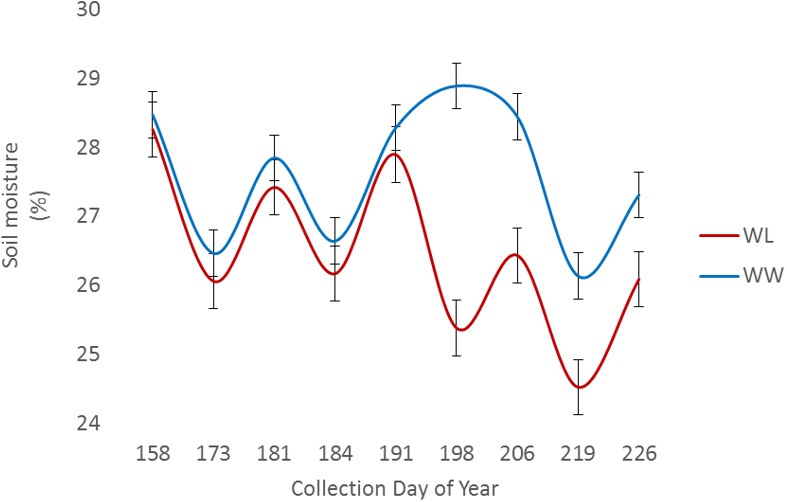
Average volumetric soil moisture content to a depth of 140 cm among sample locations for well-watered (WW) and water-limited (WL) treatments.

Two of the collections, June 16 (DOY 167) and June 30 (DOY 181), were before the irrigation treatment began on July 12 (DOY 193), while the remaining collections, July 14 (DOY 195) and July 28 (DOY 209), were post irrigation treatment initiation. The neutron moisture readings, averaged to a depth of 140 cm, showed that deviation in percent soil moisture between the irrigation treatments was not detectable until July 17 (DOY 198) (**Figure 4**). Due to the delayed soil moisture deficit, the treatment effect over time was not significant (*P*-value > 0.05) for any of the PSC derived or physiological phenotypes observed (**Supplementary Table S1**). The weather station data collected with each run also showed that the *Ta* and RAD decreased between the first run and the last, while the *Rh* increased (**Figure 3**). These environmental factors would also contribute to undetectable treatment differences over time but added value to determine how soil moisture and other environmental factors affected the sensor values.

### Cotton Entries and Sensor “Tri-Metric” Detection

**FIGURE 5:**
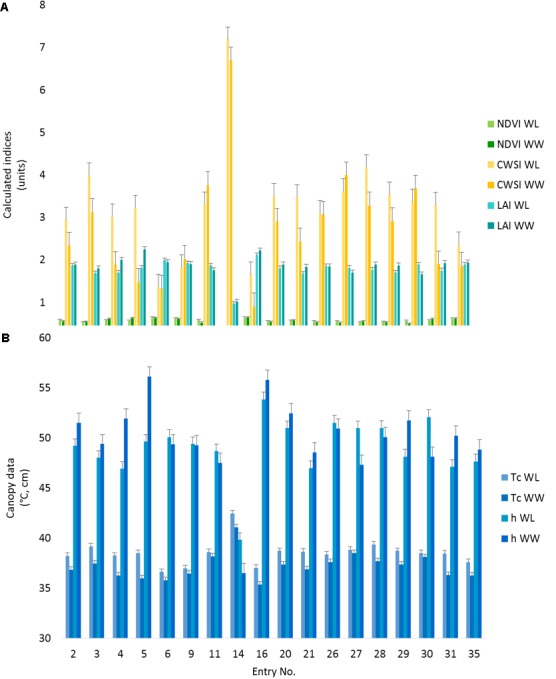
Proximal sensing cart-derived phenotypes split out by irrigation treatment for each of 18 selected cotton entries. The data represent the lsmeans of each phenotype from the repeated measure analysis for the designated treatment and cotton entry. **(A)** The NDVI, CWSI, and LAI data are presented as calculated index units with corresponding error bars. **(B)** The canopy temperature (*Tc*) and canopy height (*h*) with the corresponding error bars. Entry number 35, the local check, was used as the control for all statistical comparisons.

All the observed phenotypes differed among cotton entries (*p* < 0.05). Despite a non-significant treatment effect, the entry by treatment interactions were significant (*p* ≤ 0.05) for all the PSC phenotypes collected except for NDVI (**Supplementary Table S1**), indicating germplasm can be differentiated by the phenotypes characterized under these environmental conditions. In comparisons of the lsmeans of *Tc* with the locally adapted control (DP1549B2XF), 17 entries were different (*p* < 0.05) for the WL treatment (**Figure 5** and **Supplementary Table S2**). Of these entries, three had *Tc* values lower than the control while two (#s 6 and 16) had reduced CWSI values. For the WW treatment, entry #16, also had lower *Tc* and CWSI values than the control (*p* < 0.05) along with increased LAI values under both treatments (*p* < 0.05), indicating this entry was more drought tolerant (**Figure 5** and **Supplementary Table S2**). Entry #16 was the earliest to flower, indicating this line might be avoiding the accumulative effects of drought via faster development, whereas entry #6 flowered later, suggesting a difference in drought tolerance mechanisms (**Supplementary Table S3**). Compared to the control, Entry #14 had higher *Tc* and CWSI and lower *h*, NDVI, and LAI values (*p* < 0.05) for both irrigation treatments (**Supplementary Table S2**). This entry had poor emergence and stand establishment (**Supplementary Table S3**). Low plant stand was associated with reduced canopy cover and hence, increased soil interference in the sensors’ fields of view, meaning that reported phenotypes for this entry are likely biased.

The broad-sense heritability estimates for each PSC-derived phenotype over the four collections ranged from 0.097 to 0.574 (**Supplementary Table S4**). Estimates for NDVI and LAI were the lowest overall, ranging from 0.284–0.489 to 0.211–0.382, respectively. Estimates for *h* were the highest overall, ranging from 0.211 to 0.574. Estimates calculated from the second and third collections (DOY 181 and DOY 195) were higher and most consistent between collections (**Supplementary Table S4**). The broad-sense heritability values from the last collection (DOY 209) were the lowest, which is likely due to the increased environmental variation (*Ta, Rh*, and RAD) during that collection (**Figure 3**).

Correlations among the PSC phenotypes found significance (*p* ≤ 0.05) between all traits characterized across the irrigation treatments (**Supplementary Table S5**). The treatments were not assessed separately, because there was no treatment effect. Analysis with the physiological and growth stage measurements resulted in negative correlations between plant stand count with *Tc* (*r* = -0.695, *p* = 0.0001) and CWSI (*r* = -0.834, *p* = 0.0001), while positive correlations were found between plant stand count with *h* (*r* = 0.316, *p* = 0.008), NDVI (*r* = 0.862, *p* = 0.0001), and LAI (*r* = 0.635, *p* = 0.0001) (**Supplementary Table S4**). No significant correlations were found between PSC phenotypes and the other physiological and growth stage measurements (**Supplementary Table S5**). While not significant, a low positive correlation (*r* = 0.224, *p* = 0.062) was found between pollen sterility and first flower, indicating some of these lines might have heat avoidance mechanisms (i.e., faster growth means less accumulated stress).

**FIGURE 6:**
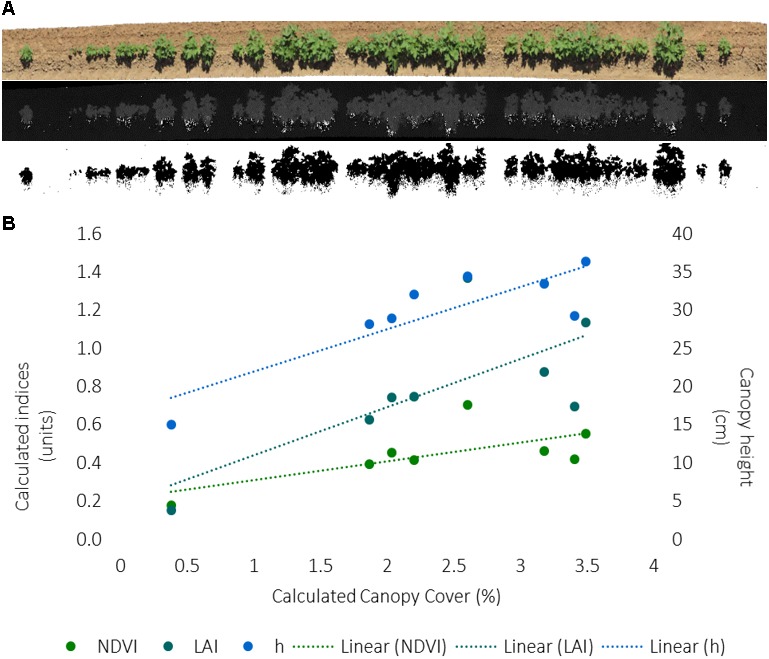
**(A)** Image workflow to calculate canopy cover from RGB images collected for each 12.1 m row of each plot, including an orthomosaic from Agisoft Photoscan software, a hue band calculated from RGB data, and the inverse of the binary image from segmentation of green hue as outputted by Fiji Image J software. **(B)** Correlation between the RGB-derived canopy cover and PSC-derived normalized difference vegetative index (NDVI), LAI, and canopy height (*h*) values for the first eight plots on one collection day. The index units are to the left, canopy height units are to the right. The dashed line indicates the excel generated trendline.

A method workflow for the image analysis is illustrated in **Figure 6A**. The captured RGB images were aligned to generate an orthomosaic in Agisoft Photoscan. The color space of the orthomosaic was converted from RGB to HSB using Fiji Image J. A binary image of plant (green) pixels was obtained by segmenting the orthomosaic using a thresholding approach with the hue band. The canopy cover was calculated as a ratio of the area of green pixels from image segmentation and the total plot area from known plot length and width.

The canopy cover analysis for the first 8-plots only, on DOY 181, and corresponding PSC and physiological measurements, found cover was positively correlated (*r* = 0.747, *p* = 0.033) with plant stand count indicating the hue threshold values accurately captured plant (green) pixels (**Figure 5B** and **Supplementary Table S6**). Similar to plant stand count, negative correlations were found between canopy cover with *Tc* (*r* = -0.789, *p* = 0.020) and CWSI (*r* = -0.788, *p* = 0.020), and positive correlations were found between canopy cover with *h* (*r* = 0.864, *p* = 0.006) and LAI (*r* = 0.721, *p* = 0.043) (**Figure 6B** and **Supplementary Table S6**). Unlike the correlations among the whole dataset, significant correlations (*p* ≤ 0.05) were found between PSC phenotypes and the physiological and growth stage measurements, particularly with first flower (**Supplementary Table S6**). This is likely due to the very small dataset, which could also be influencing the other correlations.

## Discussion

This paper describes the development and deployment protocol of a low-cost proximal sensing cart (PSC) and sensor package (SP) to characterize drought-adaptive traits in cotton germplasm grown under well-watered and water-limited conditions. The developed protocol provides several advantages to previous cart deployments including: methods to confirm geospatial alignment of data to experimental plots with the anchor targets; methods to assess quality and accuracy of the sensor value returns with the hand measurements and anchor targets; real-time environmental data to clarify environmental interactions; and a number of physiological notes to assess significant differences between genotypes. The PSC and SP reliably quantified plant characteristics related to drought tolerance in cotton by following the developed protocol. The results of this work include a new PSC design and SP, a deployment protocol for accurate data capture of canopy temperature, height, and spectral reflectance, and the identification of two cotton entries that showed improved drought-adaptive characteristics compared to the locally adapted control under irrigation treatments and changing environmental conditions.

The new PSC design is smaller than the previous model (White and Conley, 2013), which reduced the cost and weight. The narrow wheel spacing fits within the bed of a pickup truck, negating the need for light-weight trailers to transport the PSC to different field sites as previously reported. The decreased weight and extended handle allowed for easier maneuverability in the field by lifting up the back end of the cart, which permitted pivoting on two wheels during turns and negated the need for four swivel wheels as suggested by White and Conley (2013). Required improvements to the cart design include the capability to increase the cart height above 99 cm. If collections had continued to the end of the cotton season, the tops of the cotton plants would have hit the frame of the cart potentially causing damage to the plants. Motorized steering and drive control would improve the consistency at which data is collected and reduce the effort of the cart operator.

The sensor package deployed on the PSC increased the number of traits that could be observed or calculated at one time compared to the previous cart model. These traits included canopy height and NDVI which enabled the LAI to be calculated. The addition of the weather station sensor enabled a CWSI to be calculated and monitor the effects of environmental conditions on the cotton entries in real-time. The addition of these traits enables the discovery of potential mechanisms underpinning the drought-adaptive characteristics. For example cotton entry #6 had a reduced canopy temperature and CWSI compared to the control under water-limited conditions indicating a difference in stomatal regulation and water use efficiency between these two entries. The addition of more sensors with appropriate validation could further enhance the ability to determine drought tolerant mechanisms.

The broad-sense heritability estimates (repeatability) reported in this study are somewhat lower than those reported by Andrade-Sanchez et al. (2014) utilizing the high-throughput phenotyping, high clearance tractor. The reduced estimates were likely due to the earlier season measurements with smaller canopies in the first collection and the variation in the environmental conditions in the last collection. The lowest estimates calculated were from the last collection which illustrates the environmental influence on these traits. While not explored in this study, future analysis incorporating the weather data would enhance estimates of plant characteristics and improve understanding of plant response to environmental change on a small time-scale.

The canopy cover pipeline utilized in this study, while accurate, was not efficient enough to determine cover for all experimental plots in a timely fashion. Future improvements will include the addition of GPS enabled cameras with white balance calibration. This will ensure quality images and negate the need for timely spatial binning to geolocate images to plots. The processing pipeline would be further enhanced by either “batching” the HSB threshold steps or utilizing another software program that can automate the HSB threshold steps. Utilizing images in high-throughput phenotyping will increase the number of traits that can be assessed and enhance the ability to develop heat and drought tolerant cultivars in a changing environment.

While the sensor package and proximal sensing cart described in this paper are not necessarily new, the detailed protocol for deployment has not been heavily emphasized in previous cart, tractor, or UAV papers. How the HTP platforms and sensor packages are deployed can have a profound effect on the data captured. If corresponding agronomy and physiology measurements are not also recorded and taken into consideration when evaluating the captured data, researchers run the risk of developing the wrong conclusions, but this information is frequently left out of FB-HTP papers to date. As an example, the suppositions regarding the significant differences seen between lines #6, 14, and 16 would not be supported (or likely even made) had the soil moisture, real-time weather, and physiology data not been captured. Also, less emphasis was given to the CWSI values captured in this study because the corresponding weather data and soil moisture measurements did not support the captured trends. The detail described in this paper regarding the deployment of the cart is an attempt to emphasize the importance of “metadata” to interpret HTP data and set data capture standards.

The protocol developed in this study describes how to build and deploy a low-cost proximal sensing cart and sensor package for characterizing drought-adaptive traits in upland cotton. Next steps will include improving the cart design and automation of the image processing protocol. Future work will also include exploring statistical approaches that incorporate the real-time weather data to enhance trait estimates and repeatability.

## Conclusion

The proximal sensing cart design is relatively simple and inexpensive; the materials can be purchased from hardware stores and online retailers. The deployment protocol is relatively easy to execute, and the majority of software for data analysis is open source (QGIS, Python, Image J). Accurate and low-cost detection of plants in the field was achieved by utilizing a modified proximal sensing cart design and sensor package to detect spatial and temporal plant responses under drought conditions.

## Author Contributions

AT and MC optimized the PSC and deployment protocol. AT, MC, and JH performed the data collections along with the acknowledged students. AT, KT, and MC performed the data analysis. PA-S reviewed the manuscript. AT, MC, PA-S, and JH conceived the project and its components. All authors discussed the results and contributed to the manuscript.

## Conflict of Interest Statement

The authors declare that the research was conducted in the absence of any commercial or financial relationships that could be construed as a potential conflict of interest.

## Footnotes

1 ag.arizona.edu/azmet/index.html

2 www.qgis.org

## Supplementary Material

FIGURE S1 The specifications of the PSC developed for this study. The drawings were rendered in AutoCAD software where **(A)** indicates side views and **(B)** indicates top-down views of the cart.

FIGURE S2 The QGIS schematic of the experimental field showing plot boundaries (yellow), buffer rows (purple), alley ways (white), and neutron soil moisture reading locations (white circles).

TABLE S1 The outputs for each trait assessed from the repeated-measures mixed-model analysis for cart derived traits or from the mixed-model analysis from the hand measured traits. Each table contains the effects included in the model along with the corresponding degrees of freedom (DF) calculated *F* values and associated *P*-value. Model effects were considered significant at 0.05.

TABLE S2 The mixed-model DUNNETTs comparison with the local check output for each of the cart derived traits. The comparisons are split out by each cotton entry and irrigation treatment assessed. The table includes the calculated lsmeans value for the cart derived trait, the standard error (SE) and the associated *P*-value. Comparisons were considered significant at 0.05.

TABLE S3 The lsmeans and standard error (SE) derived from the mixed-model analysis for each of the hand collected traits. These values were used for the subsequent trait association analysis.

TABLE S4 The calculated broad-sense heritability estimates and standard error (SE) for each of the cart derived traits for each day of year (DOY) collection.

TABLE S5 Pearson correlation coefficients (above) and *P*-values (below) showing relationships between the cart and hand collected measurements from all collections and across treatments. Correlations were considered significant at 0.05.

TABLE S6 Pearson correlation coefficients (above) and *P*-values (below) showing relationships between the cart and hand collected measurements as well as the image derived canopy cover from the eight plots assessed across treatments. Correlations were considered significant at 0.05.
