# Supplementary figures and images for: Deploying a Proximal Sensing Cart to Identify Drought-Adaptive Traits in Upland Cotton for High-Throughput Phenotyping
Source: Front Plant Sci. 2018 Apr 23;9:507. doi: 10.3389/fpls.2018.00507 (PMC5961097; doi:10.3389/fpls.2018.00507)

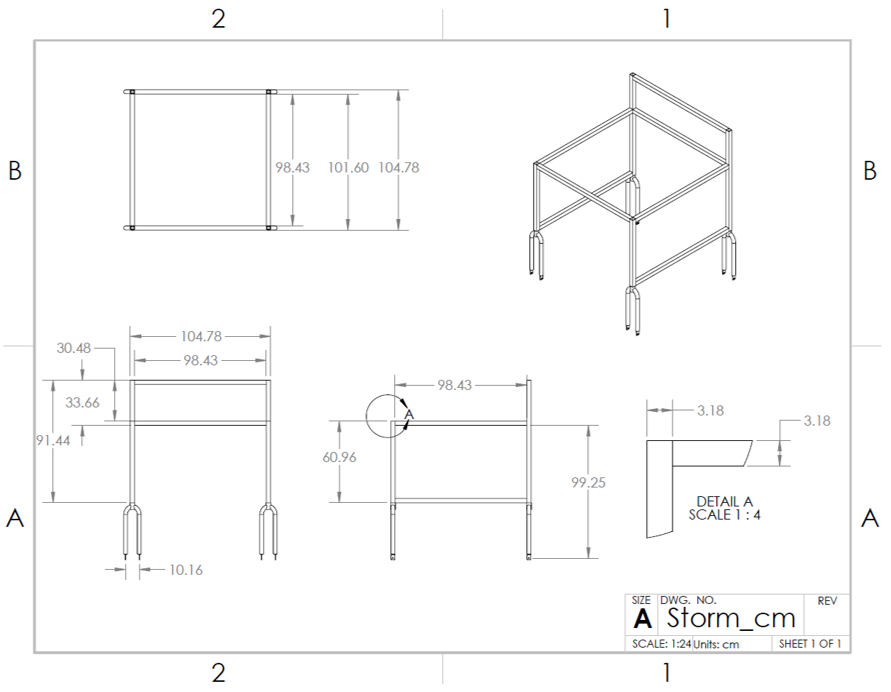

Supplement: FIGURE S1 — The specifications of the PSC developed for this study. The drawings were rendered in AutoCAD software where (A) indicates side views and (B) indicates top-down views of the cart. [file Image_1.TIF]

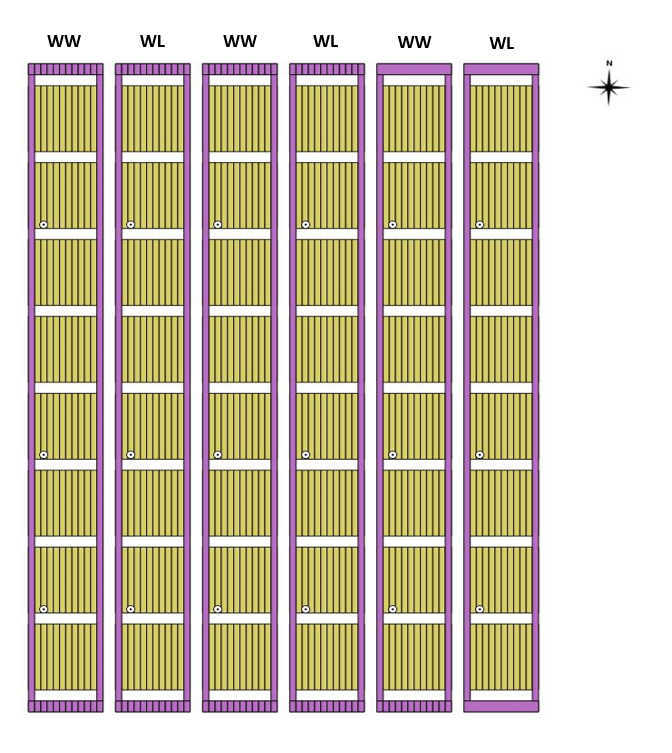

Supplement: FIGURE S2 — The QGIS schematic of the experimental field showing plot boundaries (yellow), buffer rows (purple), alley ways (white), and neutron soil moisture reading locations (white circles). [file Image_2.TIF]
