# Supplementary material for: Deploying a Proximal Sensing Cart to Identify Drought-Adaptive Traits in Upland Cotton for High-Throughput Phenotyping
Source: Front Plant Sci. 2018 Apr 23;9:507. doi: 10.3389/fpls.2018.00507 (PMC5961097; doi:10.3389/fpls.2018.00507)
Supplement: Supplementary file 4 [file Presentation_1.PDF]

Exercise G: Georeferencing Sensor Outputs
Field Phenomics Workshop, Maricopa, Arizona
Wednesday, March 18, 2015 8:30am
Instructor: Kelly Thorp (kelly.thorp@ars.usda.gov)

1. Download and install Quantum GIS version 2.6 'Brighton'
    a. Point your browser to www.qgis.org
    b. Click 'Download Now'
    c. Download the appropriate file for your operating system
    d. For Windows, select 'QGIS Standalone Installer Version 2.6 (32 bit)'
    e. Download and install
2. Install HTP Geoprocessor plug-in for Quantum GIS
    a. Open Quantum GIS Desktop
    b. Click Plugins->Manage and Install Plugins...
    c. Search 'HTP Geoprocessor'
    d. Click 'Install Plug-in'
3. Know where to find help
    a. Click Plugins->HTP Geoprocessor->Help
    b. Find a help document for all the HTP Geoprocessor tools
4. Download the files for Exercise F from the workshop webcite.
5. Create a plot map
    a. Open the file ‘plotnodes.csv’ with Excel or a text editor. Inspect.
        i. Each row provides coordinate information for one plot.
        ii. First column provides plot name or barcode. ‘Fill’ indicates buffer plots.
        iii. A set of four UTM coordinates defines four plot corners.
        iv. User must provide this coordinate file. A variety of options are possible.
    b. In Quantum GIS Desktop,
        i. Click Plugins->HTP Geoprocessor->Map Creator and inspect its user interface
        ii. Select input coordinate file: “plotnodes.csv” from above
        iii. Select output shapefile name. Navigate to your working directory and enter a name. I suggest “plotpolygons.shp”
        iv. Click ‘Run’
        v. Select the correct coordinate reference system in the pop up window. The correct one is “WGS 84 / UTM zone 12N” with Authority ID “EPSG:32612” and you can use the Filter box to find it. Click “OK”
        vi. The program will create a shapefile of plot polygons. Select “Yes” to add the shapefile to the map.
        vii. Click ‘Exit’ to close the Map Creator window.
        viii. Inspect the shapefile.
            1. Do you see the plots represented by polygons?
            2. Click the zoom button (magnifying glass with ‘+’ symbol). Can you zoom in on some plots?
            3. Right click on ‘plotpolygons’ in the layer list. Select ‘Zoom to Layer extent.’ Does the map zoom out to show the full layer?
            4. Right click on ‘plotpolygons’ in the layer list. Select ‘Open attribute table.’ What do you see? What happens if you select a feature (row) in the attribute table? Do you see the feature highlighted in the map view?

6. Preprocess a Crop Circle sensor data file from the 2012 Arizona phenotyper
    a. Open the file 'F119_2012_DOY201_1pm_CC.csv' with Excel or a text editor. Inspect.
        i. Column 1: Longitude
        ii. Column 2: Latitude
        iii. Column 7: Heading
        iv. Column 8: Sensor ID Text
        v. Columns9-13: Sensor data from one Crop Circle sensor per row – vegetation indices and reflectance measurements
    b. In Quantum GIS Desktop,
        i. Click Plugins->HTP Geoprocessor->Preprocessor and inspect its user interface
        ii. Click 'Load' and open the 'crop circle instructions.csv' file. This loads the information needed to preprocess the Crop Circle data file from above. The user must create an instruction file specific to the data format of a given data file. Here, I am providing you with an instruction file previously created for the Arizona Crop Circle data files.
            1. There are 13 total columns in the Crop Circle data file. Lines with any other number of entries will be ignored.
            2. Data from four Crop Circle sensors are recorded in the file. Thus, there are four sensor entries.
            3. Column numbers for latitude, longitude, heading, sensor ID, and data columns are given.
            4. Text for the sensor ID column is given. Information from individual Crop Circle sensors are written on different rows and thus a sensor ID is used to attribute a given line of data to a particular sensor.
            5. Easting and Northing offsets from the GPS receiver to each sensor are specified assuming the tractor is facing due north.
            6. There are offsets between the GPS receiver and the plot map in this season. These entries are specified on the bottom left. If there were no offsets between the plot map and GPS data, users can enter '0.0'
        iii. If changes were made to the instruction file, the "Save" button would permit saving those changes for future use.
        iv. Click "Run" and open the 'F119_2012_DOY201_1pm_CC.csv' file for preprocessing. The program will then calculate UTM coordinates of the receiver position, calculate sensor positions based on heading information, and write a new file to disk called 'F119_2012_DOY201_1pm_CC-preprocess.csv.'
        v. Click "OK" when finished and "Exit" the Preprocessor tool
7. Preprocess an IRT sensor data file from the 2012 Arizona phenotyper
    a. Open the file 'F119_2012_DOY201_1pm_IRT.csv' with Excel or a text editor. Inspect.
        i. Column 39: Longitude
        ii. Column 38: Latitude
        iii. Column 40: Heading
        iv. Columns3-26: Sensor data from 8 different IRT sensors on one row – mV readings and canopy temperatures
    b. In Quantum GIS Desktop,
        i. Click Plugins->HTP Geoprocessor->Preprocessor and inspect its user interface
        ii. Click 'Load' and open the 'IRT instructions.csv' file. This loads the information needed to preprocess the IRT data file from above. Because the IRT data are

recorded differently than Crop Circle, the instruction files for these two sensors are different.

1. There are 40 total columns in the IRT data file. Lines with any other number of entries will be ignored.
2. Data from eight IRT sensors are recorded in the file. Thus, there are eight sensor entries.
3. Column numbers for latitude, longitude, heading, and data columns are given.
4. There is no information for sensor ID in this file, so these are left blank. Information from individual IRT sensors are written across the columns on the same line and thus unique sensor information is specified in the data column entries.
5. Easting and Northing offsets from the GPS receiver to each sensor are specified assuming the tractor is facing due north.
6. There are offsets between the GPS receiver and the plot map in this season. These entries are specified on the bottom left. If there were no offsets between the plot map and GPS data, users can enter '0.0'

iii. If changes were made to the instruction file, the "Save" button would permit saving those changes to a file for future use.

iv. Click "Run" and open the 'F119_2012_DOY201_1pm_IRT.csv' file for preprocessing. The program will then calculate UTM coordinates of the receiver position, calculate sensor positions based on heading information, and write a new file to disk called 'F119_2012_DOY201_1pm_IRT-preprocess.csv.'

v. Click "OK" when finished and "Exit" the Preprocessor tool

8. Load preprocessed data into Quantum GIS Desktop.
   a. Open 'F119_2012_DOY201_1pm_CC-preprocess.csv' with Excel or a text editor. Inspect.
      i. Notice new columns for UTM X and Y at the receiver (UTMX-rec, UTMY-rec) and UTM X and Y at the sensor (UTMX-sen, UTMY-sen)
   b. In Quantum GIS Desktop, click Layer->Add Delimited Text Layer
      i. Browse to open the file named 'F119_2012_DOY201_1pm_CC-preprocess.csv'
      ii. File Format is 'CSV (comma separated values)'
      iii. X field: UTMX-sen
      iv. Yfield: UTMY-sen
      v. Click 'OK'
      vi. Select the correct coordinate reference system in the pop up window. The correct one is "WGS 84 / UTM zone 12N" with Authority ID "EPSG:32612" and you can use the Filter box to find it. Click "OK"
      vii. The sensor data should load into Quantum GIS as a point layer and overlay nicely on the plot map.
      viii. Convert the sensor data points to a shapefile
         1. Right click on the 'F119_2012_DOY201_1pm_CC-preprocess' layer in the Quantum GIS layer list. Select 'Save As…'
         2. Click Browse, navigate to the working directory and save the data as a shapefile named 'F119_2012_DOY201_1pm_CC-preprocess.shp'
      ix. Remove the 'F119_2012_DOY201_1pm_CC-preprocess' layer from Quantum by right clicking in the layer list and selecting "Remove"
      x. Load the 'F119_2012_DOY201_1pm_CC-preprocess.shp' file
         1. Click Layer->Add Vector Layer

        2. Browse to the shapefile that was create above
        3. Select the shapefile and "Open" it.
    c. Repeat step 8 for the IRT data file: 'F119_2012_DOY201_1pm_IRT-preprocess.csv'

9. Georeference sensor information within plot boundaries – Method 1 using default QGIS tools
    a. Click Vector->Geoprocessing Tools->Intersect
        i. Input vector layer: F119_2012_DOY201_1pm_CC-preprocess.shp
        ii. Intersect layer: plotpolygons.shp
        iii. Output shapefile: Browse to work directory and specify the file as 'F119_2012_DOY201_1pm_CC-preprocess-intersect.shp'
        iv. Select 'Add result to canvas'
        v. Click 'OK' – It will take some time to run.
    b. Inspect the resulting map of sensor data points. Notice this approach removes the points that fall outside of the plot boundaries.
    c. Inspect the Attribute Table of the resulting shapefile. Do you find the appropriate plot name (barcode) specified for each sensor data point?
    d. Repeat step 9 for the IRT data file: 'F119_2012_DOY201_1pm_IRT-preprocess.shp'

10. Georeference sensor information within plot boundaries – Method 2 using HTP Geoprocessor
    a. Click Plugins->HTP Geoprocessor->Geoprocessor
        i. Select Processing Objective: Add attributes of polygons to points
        ii. Select Base Layer: F119_2012_DOY201_1pm_CC-preprocess
        iii. Select Layer to Process: plotpolygons
        iv. Select Data Fields to Process: BARCODE
        v. Click 'Run' – It will take some time to run.
    b. Inspect the Attribute Table of 'F119_2012_DOY201_1pm_CC-preprocess.shp'. Do you find the appropriate plot name (barcode) specified for each sensor data point? Notice that the second approach appended the barcode information to the sensor data shapefile directly, without creating a new shapefile and without deleting the data points falling outside the plot boundaries.
    c. Repeat step 10 for the IRT data file: 'F119_2012_DOY201_1pm_IRT-preprocess.shp'
