# Supplementary material for: Deploying a Proximal Sensing Cart to Identify Drought-Adaptive Traits in Upland Cotton for High-Throughput Phenotyping
Source: Front Plant Sci. 2018 Apr 23;9:507. doi: 10.3389/fpls.2018.00507 (PMC5961097; doi:10.3389/fpls.2018.00507)
Supplement: Supplementary file 5 [file Presentation_2.PDF]

Exercise H (field): Introduction to Image Analysis for Phenomics using Image J
Wed, 13:00

Jeff White, Steve Welch, Andy French and Matt Conley

Objectives

I. Introduce basic concepts of image analysis using the open source software ImageJ
   A. Image properties
      1. Image type and resolution
      2. EXIF data
   B. Basic manipulation
      1. Crop
      2. Segmentation
   C. Analysis and output of results
II. Four exercises
   A. Canopy cover: Manual segmentation and analysis
   B. Canopy cover: Batch processing
   C. Seed count and morphology: Batch processing
   D. Infrared thermometry: Manual segmentation

Activities

I. Install and launch ImageJ version 1.48
   A. Navigate to http://imagej.nih.gov/ij/download.html
      1. Select the correct version for your computer.
      2. Download and install. You must have Administrator privileges for your computer.
II. Canopy cover: Manual segmentation and analysis

"Canopy cover" is simply the portion of the total area that is covered by plant material when viewed from overhead (nadir). The value may be reported as a fraction or percentage. Canopy cover is strongly related to leaf area index up to canopy closure. Our challenge here is to separate pixels that represent plant tissue from pixels that represent soil.

   A. Open a single image of camelina (an oil seed crop in the Brassicaceae).

1. Start ImageJ
2. From the tool bar, select File→Open
3. Navigate to the folder where you have placed the exercise material
4. Open the folder "`Canopy_images`"
5. Select the image "`camelina2010_061_#7473_16-nha.tif`"
6. The image should appear in a new window
7. Inspect the window: At the top left, one sees information about the image:
   a) It has three channels (Red, Blue and Green). We are at the red channel, although the displayed image is for all three channels.
   b) The size is 3456 x 2304 pixels (ignore dimension in inches or cm).
   c) The image is in a 16-bit format, and the file size is 46MB.

B. To "segment" the image between soil and canopy, we will use the Hue-Saturation-Brightness (HSB) color space

1. Run: Image→Type→RGB color.
2. Run: Image→Type→HSB-Stack. The image has been converted to three gray-scale stacks within an image that represent Hue, Saturation and Brightness. The slide bar at the bottom allows you to view the three coordinates of the HSB color space.
3. Run: Image→Stacks→ Stacks to Images. This creates separate gray-scale images contained in a stack.
4. Click on the Hue image (check label in upper leftto make it active. Run: Image→ Adjust→ Threshold. The Hue image should appear with canopy showing as red. Set the lower and upper threshold limits to 35 and 125.
5. Hit Apply, and OK for the next screen.
6. Close the Threshold window.
7. Run: Process→ Binary → Convert to Mask
8. Run: Edit → Invert. Plant tissue should now be black. Soil is white.
9. Select the correct measurement by running: Analyze → Set Measurements and then check the box for Area fraction. Select OK to close.
10. Run: Analyze → Measure. A box should appear with a value for %Area of 59.996.
11. Close the box.

12. To check whether we accidentally include soil or removed too much plant, let's return to the original image. It should be open as the only color image. You can close Saturation and Brightness. If it isn't open, please re-open from File→ Open.

13. Click on the original image to make it the active image.

14. Run: Image → Overlay → Add Image. In the menu box, select "Hue" as the image to add. Set Opacity to 25% and select "OK."

15. The color image should now appear with soil somewhat grayed out. To see where the process has incorrectly classified pixels, zoom in using the "+" key (zoom out with "-").

16. That was fun but a bit error-prone. Let's close the images and try something better.

III. Batch processing

Analyzing single images with menus commands is useful for testing new methods but is impractical for processing large numbers of images. ImageJ allows users to write macros that can process multiple image files in a directory. We demonstrate a macro that estimates canopy cover.

A. Run: Plugins → Macros → Run. A file selection box should appear. Navigate to Image_analysis_exercises and select the file *Canopy_cover_via_HSB_ranges.ijm*

B. A new folder/file selection box should appear that says "Choose SOURCE Directory." Navigate to the folder Image_analysis_exercise and select the folder "Canopy_images." ***Without opening the folder***, click on "Select".

C. A second folder/file selection box should appear that says "Choose DESTINATION Directory." Navigate to the folder Image_analysis_exercise and select the folder "Canopy_images_analysed." **Without opening the folder**, click on "Select".

D. The macro starts immediately. It displays information on which file is being processed and how much memory is being used. The directory has nine images. As the processing advances, a window labeled "Results" is updated.

E. When the processing is finished, the Canopy Cover Results window closes, but a copy has been written to the destination folder. Navigate to that folder (using ImageJ or Windows Explorer or equivalent) and open the file.

F. The macro also produces binary images showing the effects of the segmentation. Presence of isolated small speckles suggests that some non-canopy material was mis-classified.

IV. Counting seed

This exercise shows how objects can be counted using ImageJ. Although not a field activity, counting seed in a sample is often a time-consuming step in post-harvest analyses. The method can also be extended to measure the size of individual seed or characterize variation in color.

A. Inspect the source images:

1. (Close any open images or boxes)

2. Run: File → Open . Navigate to the folder *Image_analysis_exercise/ Source_seed_images*

3. Select *Wheat_sample.jpg*. An image of wheat seed on a black background should appear. This image has 50 seed. Note that some are touching.

4. This image was obtained using a Fujitsu ScanSnap SV600, which will be on display in the lecture hall.

5. View information on the image by typing “i”. A window appears telling us that the resolution is 200 dots per inch.

6. Close the box and the image.

B. Analyze the images

1. Using the ImageJ menu, run: Plugins → Macros → Run. This will open a file navigation dialog box.

2. Navigate to the folder *Image_analysis_exercise* and select the macro *baseline_seed_counting_macro_v2.1.ijm*

3. Run the macro by clicking “Open”.

4. A new file navigation box will open. Following the prompt “Choose SOURCE directory” (top left of box), select the folder *Source_seed_images*. (Select the directory name; not the images themselves.)

5. A second, similar navigation box will appear with the prompt “Choose DESTINATION directory” (top left of box). Select the folder *Destination_seed_counts_w_images_and_data*.

6. The macro will begin analyzing the images and outputting two summary images and a data file for each input image of seed.

7. As each seed image file is processed, a series of boxes with data will appear briefly.

8. At the completion of processing all of the images, the summary results will be displayed along with a message box.

C. Viewing the outputs

1. You can view the summary results for each image here (you may have to move the message box), but a better way is to view the resulting files and images.

2. Click on "OK" to close the macro.

3. Navigate to the destination directory to view the macro outputs.

4. Open the file pinto2_blk_man2.jpgoutline.png. At the bottom, you will see that the last seed counted was number 100, so the macro worked pretty well.

5. Open the file Black_eyed_pea.jpegoutline.png

   a) What went wrong here?

6.

D. Understanding the outputs of the seed counting macro

1. File naming

2. Output files corresponding to a single source image of seed are identified with a prefix consisting of the entire input filename, sometimes including the extension. Thus, for an input of Wheat_sample_2.jpg, the macro creates files labeled:

   a) Wheat_sample_2.jpgresults.txt

   b) Wheat_sample_2.jpgoutline.png

   c) Wheat_sample_2.jpgbinary.png

3. The summary table of results is stored in summary.txt as tab-delimited data. The file can be easily viewed in a spreadsheet tool (e.g., Open Office or MS Excel).

4. Contents of output data files:

5. Numerical results for one image file of seed (e.g., *Wheat_sample_2.jpgresults.txt*)

   a) This file contains the data for individual objects recognized in the binary image plus three fields (columns) to facilitate diagnosing performance problems. The individual fields are explained in Table S1.

6. Outline for a single image file (e.g., *Wheat_sample_2.jpgoutline.png*)

7. Binary for a single image file (e.g., *Wheat_sample_2.jpgbinary.png*)

8. Summary results for all files (*summary.txt*)

a) This file contains totals or means for the set of images processed. The individual fields are explained in Table S1. In most cases, only the source file ("Slice") and count are of interest.

V. Canopy temperature: Viewing JPEG EXIF data & manual segmentation and analysis

We will work from an image from the FLIR camera, which you saw demonstrated in the field Monday PM. The temperature range is shown indicated by the bar on the right using the usual convention of blue is cold and red is hot.

A. EXIF information.

Image files in JPEG format often carry a large amount of useful data in the EXIF header. With ImageJ (and many other tools), you can view this information to learn when the image was taken, what camera type and settings were used (focal length, aperture, etc.), and other information that may be valuable in post-processing.

1. From the tool bar, select File→Open
2. Navigate to the folder "`Infrared_thermometry`" and open.
3. Select the file "`IRT_FLIR0071.jpg`"
4. The image should appear in a new window
5. With the cursor over the image, type "I".
6. A window should appear with the EXIF information for the image.
7. Scroll down to view types of data
    a) The picture is relatively small/low resolution: 640 x 480 pixels.
    b) For example, there are 13 variates for geocoordinates and elevation.
8. Close the Info window.

B. Let's try to segment the image so we can get temperature data only for the crop surface.

1. Image → Adjust → Color
2. Change Threshold color to White
3. Change Color space to RGB
4. The Thresholding method is set to Default. The white regions are being excluded. We're still getting too much soil.
5. From the temperature scale at the right, we need to eliminate the hottest parts of the image shown in red. Set the limits to 0 and 255. More soil is eliminated.
6. Set the lower limit for Green to 0. Then slide the upper limit to see if you can remove more soil.

7. Set the lower limit for Blue to 0. Then slide the upper limit to see if you can remove more soil.

8. The results are not very satisfactory. We have two fundamental problems:

   a) Since parts of the soils and foliage are at the same temperature, we need additional information to separate foliage from soil. If we had a conventional image of the canopy, we could use the canopy cover to create a mask and then mask out soil from the image.

   b) While the Blue-to-Red temperature scale is attractive visually, it is hard to analyze directly as temperature. A simple gray-scale might work better. Also, rather than allow the camera to set the end-points of the scale, one might want to fix the temperature range when capturing the images.

Table S1. Explanations of fields in tabular output result for a single image file (e.g., *Wheat_sample_2.jpgresults.txt*). Units of measurement are based on pixels unless a scale is set within ImageJ. A = area and P = perimeter.

**Variable**	**Description**
Area	Area (A) of a single seed or other object[1].
XM, YM	The average of the x and y coordinates all pixels in an object.
Perim.	The perimeter (P) of the object.
Circ.	Circularity defined as $C = 4\pi \, (A/P^2)$
Feret	The Feret diameter is the longest distance between any two points along the object's boundary, also known as the maximum caliper diameter.
FeretX, FeretY	The starting coordinates of the line representing the Feret diameter.
FeretAngle	The angle of the Feret's diameter in units of degree-angle (0 to 180°).
MinFeret	The minimum distance between any two points along the object's boundary.
AR	The aspect ratio of the object's fitted ellipse.
Round	The roundness of the object defined as R = Major axis/Minor axis
Solidity	The solidity of the objects defined as S = Area/Convex area
(file name)	The file name of the binary image (given as a column header)
Proc_time_sec	Total processing time of the image. Provided to assist de-bugging.
Memory_usage	Memory used during image processing. Provided to assist de-bugging.

[1] In ImageJ terminology
